# State Work Well Done

**Published:** 1898-03

**Authors:** 


### STATE WORK WELL DONE.

The excellent work done in veterinary sanitary work in Pennsylvania and Minnesota, under the direction of Veterinarians Pearson and Reynolds, teaches the value of skilled service in fields of this character. Both well adapted by education, training, and special natural ability for this work, the volume of work accomplished and the increased knowledge conveyed to all those engaged in animal industry, as well as the relations of the layman to this subject, and his indirect interest in its outcome and importance, have advanced in these States the appreciation of the value of this work ten years. Other States might well consider the great advantages to be derived by the selection of specially equipped men for such service, and thus insure safeguards to those engaged in animal husbandry representing a saving of millions of dollars annually, not to speak of the untold benefits to every man, woman and child in purer and better food-products and the great increase in the consumption of the same.

---

The new dress and better paper of our esteemed contemporary, the *American Veterinary Review*, as it appears with the February number, add much to its attractiveness. Following in the lead of the Journal, the *Review* is sent out in envelopes in place of wrappers, which makes it more presentable and better protects its edges and cover-pages than when wrapped up tightly.

---

Secretary Wilson is urging Congress for a special appropriation of $100,000 to extend the meat-inspection service in order to fulfil the demands of Europe for our meat-products. He also urges an appropriation of $100,000, to be expended in the serum-treatment of hog-cholera, and estimates the country's losses last year from this disease at $100,000,000. Every veterinarian should lend his influence in aiding the Department of Agriculture in extending the work of inspection. How far the Government should do this inspection-work without a proportion of the cost being imposed upon those directly benefited is a serious problem, and whether the production of serum, after the experimental stage, for gratuitous distribution to one class of people is the province of our Government is very doubtful indeed.

---

SAVANNAH, Ga., through its City Council, has just passed an ordinance providing for a special annual tax of thirty dollars a year on veterinary surgeons. We believe that this is the only city in the United States that imposes such an unjust tax or has taken a commercial view of what is recognized to-day all over our land as a profession. We would like to ask of the authorities of that city, Do you tax your dentists and physicians?

---

THE death of Dr. D. P. Frame, of Colorado Springs, is a keen loss to the profession of an honored member, an earnest worker, and one who was progressive in spirit and aggressive in labor. In the prime of life, and when his labors were yielding the most promising results, his sudden death reveals the uncertainty of life and teaches us how mysterious are the plans and workings of Divine Providence.

---

THE Missouri Experiment Station has added, by experimental trials, to the now generally accepted relation of value between broad-tired wagons and good roads. On macadamized street and gravel roads, dirt-roads in all conditions, except when soft or sloppy on the surface, underlaid by hard roadbed, or when the mud was very deep and sticky, the broad tires showed materially less draught, while the roads were improved and kept comparatively free from deep ruts. A six-inch tire was found to be the best for the road, meadows, pastures, stubble land, corn land and ploughed ground in every condition, from dry, hard and firm to very wet and soft. Better roads mean better horses, more driving, and a larger field of service for horses.

---

THE meeting of the Pennsylvania State Veterinary Medical Association at Philadelphia, on March 8th and 9th, will be an important gathering among the Keystone State veterinarians. The programme, with a varied series of attractions, will be full of interest to every active veterinarian. The committee reports promise to be of unusual interest; special features of operations and an illustrated lecture by Bacteriologist Ravenel, of the State Live-stock Sanitary Board. A dinner to attending veterinarians by Dr.

Leonard Pearson will be a pleasant feature. A midday luncheon to all visitors and members each day by the City of Brotherly Love veterinarians will add to the comfort and pleasure of all who may favor the Keystone convention with their presence and interest. A cordial and fraternal invitation is extended to veterinarians in our sister border States.

